# Giving and receiving thanks: a mixed methods pilot study of a gratitude intervention for palliative patients and their carers

**DOI:** 10.1186/s12904-023-01172-x

**Published:** 2023-04-26

**Authors:** Mathieu Bernard, Emmanuelle Poncin, Emilie Bovet, Emmanuel Tamches, Boris Cantin, Josiane Pralong, Gian Domenico Borasio

**Affiliations:** 1 Palliative and Supportive Care Service, Lausanne University Hospital and University of Lausanne, Av. Pierre-Decker 5, Lausanne, CH-1011 Switzerland; 2 Haute École de Santé Vaud (HESAV), Haute École Spécialisée de Suisse Occidentale (HES-SO), Lausanne, Switzerland; 3 Palliative Care Center, Fribourg Hospital, Fribourg, Switzerland; 4 Rive Neuve Foundation, Blonay, Switzerland

**Keywords:** Gratitude, Palliative care, Quality of life, Quality of relationship, Psychological distress, Mixed-methods

## Abstract

**Background:**

Psychological research examining the nature and workings of gratitude has burgeoned over the past two decades. However, few studies have considered gratitude in the palliative care context. Based on an exploratory study which found that gratitude was correlated with better quality of life and less psychological distress in palliative patients, we designed and piloted a gratitude intervention where palliative patients and a carer of their choice wrote and shared a gratitude letter with each other. The aims of this study are to establish the feasibility and acceptability of our gratitude intervention and provide a preliminary assessment of its effects.

**Methods:**

This pilot intervention study adopted a mixed-methods, concurrent nested, pre-post evaluation design. To assess the intervention’s effects, we employed quantitative questionnaires on quality of life, quality of relationship, psychological distress, and subjective burden, as well as semi-structured interviews. To assess feasibility, we considered patients and carers’ eligibility, participation and attrition rates, reasons for refusal to participate, appropriateness of intervention timeframe, modalities of participation, and barriers and facilitators. Acceptability was assessed through post-intervention satisfaction questionnaires.

**Results:**

Thirty-nine participants completed the intervention and twenty-nine participated in interviews. We did not find any statistically significant pre/post intervention changes for patients, but found significant decrease in psychological distress for carers in terms of depression (median = 3 at T0, 1.5 at T1, *p* = .034) and total score (median = 13 at T0, 7.5 at T1, *p* = .041). Thematic analysis of interviews indicates that overall, the intervention had: (1) multiple positive outcomes for over a third of interviewees, in the form of positive emotional, cognitive, and relational effects; (2) single positive outcomes for nearly half of interviewees, who experienced emotional or cognitive effects; (3) no effect on two patients; and (4) negative emotional effects on two patients. Feasibility and acceptability indicators suggest that the intervention was well received by participants, and that it should adopt flexible modalities (e.g. writing or dictating a gratitude message) to ensure that it is feasible and adapted to individual needs and preferences.

**Conclusions:**

Larger scale deployment and evaluation of the gratitude intervention, including a control group, is warranted in order to have a more reliable evaluation of its effectiveness in palliative care.

**Supplementary Information:**

The online version contains supplementary material available at 10.1186/s12904-023-01172-x.

## Background

Whilst psychological interventions in palliative care have concentrated on the treatment of psychopathologies such as depression and anxiety, the past two decades have witnessed mounting interest in the positive psychological factors that can improve the quality of life of palliative patients and their family carers. One of these positive determinants is gratitude. The concept usually refers to either a personality trait, i.e. a grateful disposition that is “part and parcel of the good life” and helps people to perceive and appreciate the positive in their lives [1, 2]; or to a positive, other-oriented emotion of appreciation for a tangible or intangible “gift” from someone or something else [3–5]. In the general population, gratitude – as a trait or emotion – has been associated with greater wellbeing and less psychological distress [2, 6].

These associations have underpinned the development of interventions designed to foster gratitude, usually through journaling, with participants listing the things they are grateful for, or behavioural expression of gratitude, whereby participants write and share gratitude letters with close ones [2, 6, 7]. Meta-analyses have reported that in the general population, gratitude interventions could lead to significant improvements in wellbeing, happiness, life satisfaction, and positive affect, and to small but significant decrease in depression and anxiety [7–9]. Recent systematic reviews have also suggested that gratitude interventions may improve some aspects of physical health, such as subjective sleep quality [10, 11].

If the bulk of the research on gratitude has initially been undertaken in non-clinical contexts, notably involving college students [7], some early experimental studies have focused on clinically relevant populations [12]. In the field of oncology, several studies on gratitude have been conducted in the past decade. Gratitude has been associated with positive emotions, perceived social support, posttraumatic growth, and reduced psychological distress in breast cancer patients [13, 14]. Studies also found that gratitude interventions could improve daily psychological functioning, perceived support, use of adaptive coping, and lessen fear of death and decline in positive affect in women with breast cancer [15, 16]. A recent randomised controlled trial with advanced cancer patients in Indonesia showed that a week-long mindful gratitude journaling intervention – i.e. daily gratitude listing and meditation on the objects of gratitude – helped patients experience less anxiety and depression and greater spiritual wellbeing than those who took part in a routine journaling intervention [17].

By contrast, few studies have considered gratitude in palliative care contexts [18, 19]. Yet gratitude is relevant to palliative care for two main reasons. Firstly, it can be conceptualised as an “inherently dyadic” social emotion, based on social concerns and serving a social function [20, 21]. This is important, since family and social relations have been identified as the primary factors contributing to meaning in life and quality of life for palliative care patients [22]. Secondly, experiences of loss, whether real or imagined (e.g. losing a relative, reflecting on one’s own death), were found to enhance gratitude, increasing people’s appreciation of what they still have [23, 24]. Such experiences are all too familiar to people with life-threatening illnesses, who are likely to undergo multiple losses throughout the course of their illness, e.g. of physical functions, independence, sense of identity, and future, and to reflect on their own mortality [25]. These considerations prompted us to investigate gratitude in palliative care patients in an exploratory study, which found that gratitude was correlated with better quality of life, greater posttraumatic growth, and less psychological distress [26, 27].

On the basis of these findings, we designed and piloted a gratitude expression intervention in palliative care for patients and a carer of their choice. The novelty of the intervention lies in reciprocal expressions of gratitude by dyads of participants (a patient and a carer), who wrote a gratitude letter to each other and shared it. We hypothesised that our gratitude intervention – through which participants were asked to reflect on what they were grateful for, express their gratitude, and receive the gratitude of others – might help people to gain greater awareness and appreciation of the positive in their lives. We also hypothesised that involving patient-carer dyads might lead to greater impacts on interpersonal relationships than interventions with single participants. The aims of this study are to establish the feasibility and acceptability of our gratitude intervention, and to provide a preliminary assessment of its effects.

## Methods

### Participants

This study was conducted in three partner palliative care institutions in French-speaking Switzerland: Rive-Neuve Foundation, Fribourg Hospital, and Lausanne University Hospital. It was approved by the Ethics Committee of the Canton of Vaud. Recruitment took place between November 2018 and March 2020. We initially aimed to recruit 30 patient-carer dyads, following good practice recommendations for pilot studies [28, 29] – with Whitehead and colleagues recommending a pilot sample size of 25 for small standardised effect sizes (0.2) for a main trial designed with 90% power and two-sided 5% significance, for instance [30]. To do so, care teams in our partner institutions identified eligible patients using the following criteria: (i) age > 18, (ii) progressive illness with reduced life expectancy, (iii) enrolled in palliative care, (iv) clinical state enabling the person to take part in research, (v) no total social isolation, (vi) no significant cognitive or psychiatric disorders, and (vii) no severe communication problems. A member of the research team informed eligible patients about the study, in writing and orally, and asked prospective participants to identify a carer they wished to perform the intervention with. Carers’ eligibility criteria were: (i) age > 18, (ii) no significant psychiatric or cognitive disorders, and (iii) no severe communication problems. We informed carers about the study, then collected written informed consent from both participants.

### Study and intervention design

This two-phase pilot intervention study adopted a mixed-methods, concurrent nested, pre-post evaluation design – characterised by a unique data collection phase, a predominance of quantitative methods, and the deployment of qualitative data collection and analysis methodologies to make sense of and enrich statistical findings [31]. To assess the intervention’s effects, we employed quantitative questionnaires on four indicators, namely quality of life, quality of relationship, psychological distress, and subjective burden. We also used semi-structured interviews to explore areas beyond those considered through questionnaires and access participants’ narrated experiences of the intervention.

To assess feasibility, we considered: 1) patients and carers’ eligibility rates (the proportion of patients/carers who met the study’s formal eligibility criteria), participation rates (the proportion of informed patients/carers who gave their written consent) and attrition rates (the proportion of patients/carers who formally agreed to participate but did not complete the study), 2) reasons for refusal to participate, 3) appropriateness of intervention timeframe, 4) specific modalities of participation, and 5) barriers and facilitators in undertaking the intervention. These criteria are aligned with the British National Institute for Health Research’s guidance for feasibility studies [32]. Acceptability was assessed through post-intervention satisfaction questionnaires in the second study phase.

In the first study phase, we collected baseline quantitative data on our four indicators, then asked participants to perform the gratitude intervention within a 7 to 10-day period. To do so, we provided them with short instructions on writing their gratitude letter (as detailed in additional file 1) and asked them to share their letter with each other in a way of their choosing (e.g. reading it to each other, sending it). Five to 10 days after completing the intervention, we collected post-intervention quantitative data on the four indicators. We planned this second research appointment during our first encounter with participants to encourage them to respect the study timeframe. We then conducted semi-structured interviews five to 10 days afterwards.

As recruitment proved difficult and the research process overly lengthy, we implemented a second study phase (after recruiting 11 patients in phase 1). We shortened quantitative assessments in an attempt to improve feasibility (e.g. at T0, from 61 to 7 items for patients), which enabled us to undertake post-intervention quantitative and qualitative assessments during a single appointment. The study was opened to patients wishing to participate on their own, without involving a carer, or wishing to designate a healthcare professional as the recipient of their gratitude message, instead of a family carer. We also proposed alternatives to letter writing, i.e. dictating or audio-recording messages of gratitude.

### Instruments and analysis

In the first phase of the study, we used standardised and validated questionnaires to assess: (1) quality of relationship – Couple Satisfaction Index, CSI-4 [33] and Positive–Negative Relationship Quality scale, PN-RQ [34]; (2) quality of life – McGill Quality of Life Questionnaire – Revised, MQoL-r, with patients [35] and Quality of Life in Life-Threatening Illness—Family Carer Version 2, QOLLTI-F V2, with carers [36]; (3) psychological distress – Hospital Anxiety and Depression Scale, HADS, in patients [37, 38] and Brief symptom inventory-18, BSI-18, in carers [39]; and (4) subjective burden – Self-perceived Burden Scale, SPBS, with patients [40, 41] and Burden Scale for Family Caregivers—short version, BSCF-s, with carers [42]. To translate questionnaires unavailable in French (i.e. CIS-4, PN-RQ, and SPBS) and ensure their cultural adaption, we followed instructions from the manual for cross-cultural adaptation and psychometric validation [43].

In the second study phase, we selected one to two items per indicator – quality of relationship: last two items of CSI-4; quality of life: single question about global, subjective quality of life from the MQoL-r and QOLLTI-F V2; psychological distress: two items of the Integrated Palliative Care Outcome Scale (IPOS) French version [44]; and subjective burden: last item from the BSFC-s and the SPBS for carers and patients respectively. We also added one item to assess gratitude from the Gratitude Adjective Checklist (GAC) [45], as well as Likert scale items at T1 to assess participants’ satisfaction with the intervention (e.g. whether they found the intervention beneficial, whether it improved their quality of life). Statistical analyses include descriptive statistics for socio-demographic and medical data and the four indicators, and comparison of the four indicators between T0/T1 using Wilcoxon matched-pairs signed rank test. We used IBM SPSS Statistics version 23. As this was an exploratory study, we did not perform Bonferroni corrections.

Qualitative data was generated through semi-structured interviews (as detailed in additional file 2), which explored participants’ conception of gratitude and their experience of the intervention. Interviews were audio-recorded and transcribed verbatim. We performed an inductive thematic analysis of interview transcripts [46], seeking to understand the effects of the gratitude intervention and identify the barriers and facilitators encountered by participants. Two researchers independently coded 10 randomly selected interview transcripts, compared and agreed on initial transcripts coding. The entire dataset was then coded and, through constant comparison, chosen themes and subthemes were fine-tuned in the light of new data.

## Results

Thirty-nine individuals completed the intervention and quantitative assessment of the four indicators. They include 23 patients (70% females, mean age 65, 70% cancer diagnosis) and 16 carers (81% females, mean age 59, 50% spouse/partner), whose socio-demographic and/or medical data are shown in Tables 1 and 2 respectively. Twenty-nine people participated in semi-structured interviews (18 patients, 11 carers).

**Table 1:** Patients’ socio-demographic and medical data (*n* = 23)

Variables	Value	(%)
Age		
Mean	65.3	
Standard deviation	12.1	
Sex		
Male	7	30.4
Female	16	69.6
Nationality		
Swiss	23	100
Mother tongue		
French	16	69.6
Spanish	4	17.4
Other	3	13
Marital status		
Single	5	21.7
Married/ or registered partnership	9	39.1
Divorced or separated	6	26.1
Widow	3	13
Education		
Primary / secondary school	7	30.4
Professional school	6	26.1
Gymnasium / High school	1	4.3
Technicum/HES/HEG/HEP/Normal School/ETS	3	13
University/EPFL/ETH	6	26.1
Primary diagnosis		
Cancer	16	69.6
Other	7	30.4

**Table 2:** Carers’ socio-demographic data (*n* = 16)

Variables	Value	(%)
Age		
Mean	59.3	
Standard deviation	13.1	
Sex		
Male	3	18.7
Female	13	81.3
Nationality		
Swiss	16	100
Mother tongue		
French	15	93.7
German	1	6.3
Marital status		
Single	3	18.7
Married	10	62.5
Divorced or separated	2	12.5
Registered partnership	1	6.3
Education		
Primary / secondary school	2	12.5
Professional school	3	18.7
Gymnasium / High school	2	12.5
Technicum/HES/HEG/HEP/Normal School/ETS	2	12.5
University/EPFL/ETH	7	43.8

### Intervention feasibility

**Fig. 1:**
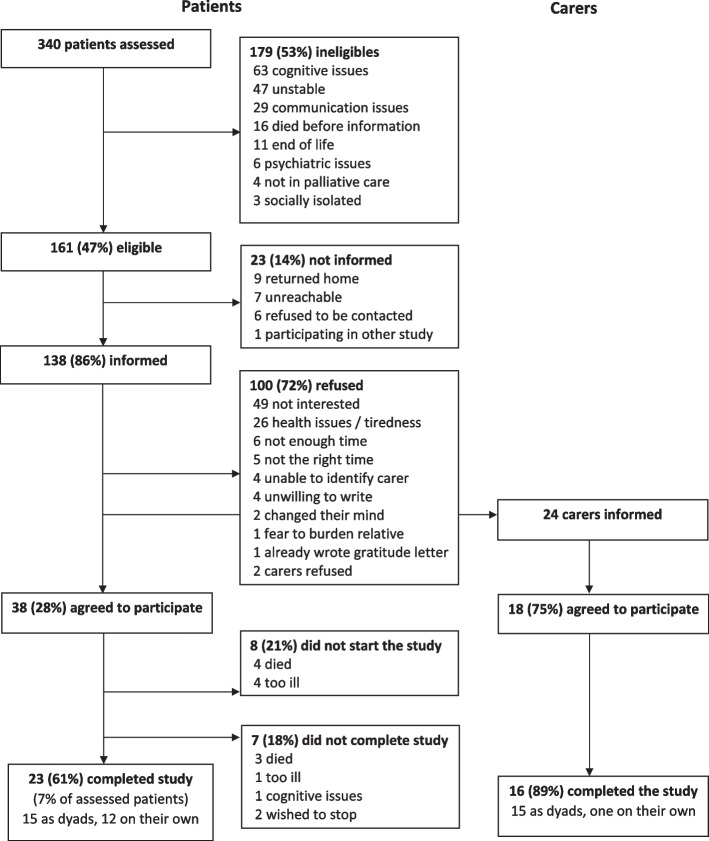
Recruitment flowchart

As shown in Fig. 1, 340 patients were assessed for eligibility, 161 of whom were formally eligible (patients’ eligibility rate: 47%). We informed 138 patients, 38 of whom formally agreed to participate (patients’ participation rate: 28%). Eight patients were unable to start the study, and another seven dropped out during the study (patients’ attrition rate: 39%). Overall, 23 patients completed the study – 11 in phase 1, 12 in phase 2. One hundred patients refused to participate, mainly because they were not interested (e.g. “I don’t want to dwell on everything”; “my participation in the illness […] does not warrant any gratitude”) or experienced health issues and fatigue. Four people further expressed a clear dislike in writing (“it’s a punishment”). It should also be noted that patients’ participation rate was 24% (19/90) in phase 1 against 40% (19/48) in phase 2, whilst patients’ attrition rate went from 42% (8/19) in phase 1 down to 37% (7/19) in phase 2. In turn, all the carers identified by patients were formally eligible (carers’ eligibility rate: 100%). We informed 24 carers about the study, 18 of whom were included (carers’ participation rate: 75%). Sixteen completed the study and two dropped out (carers’ attrition rate: 11%).

Twenty-three participants (17 patients, 6 carers) provided us with quantitative feasibility data on timeframe and transmission modalities. On average, participants took 1h10 to write their letters (min: 10mns, max: 4h30). All respondents found that the time allocated to the intervention (7 to 10 days) was sufficient. Transmission modality were heterogeneous: eight people exchanged their letters (by post or by hand), four read it aloud to the other, three read together silently, three were hesitant about sharing their letters, and one person wrote after the recipient (patient) had passed away. Data is missing for four participants. Overall, 15 patients and 15 carers completed the study as part of a dyad, and 8 patients completed it on their own, as did a carer, whose relative completed T0 only. Whilst the majority of participants (32/39; 82%) wrote their letter themselves, two were helped by their relative, three dictated their letter to a researcher or volunteers, and two audio-recorded their message with a researcher.

Thematic analysis of interview transcripts enabled us to identify three main intervention facilitators: (1) awareness of one’s own thoughts and feelings (“it seemed to me quite clear in fact, what I was grateful for”), which led some participant to experience letter-writing as self-evident (“the words were nearly ready to come out”); (2) ease with writing (“I write quite easily”); and (3) gratefulness in everyday life (“it’s an attitude that I cultivate”), which some participants experienced particularly strongly since the illness (“I’ve felt it [gratitude] more personally those past few years because of the illness”). Participants faced four main barriers: (1) written expression difficulties (the letter was “torn and torn again”); (2) physical difficulties (“I was very very tired”); (3) unprocessed memories (“there are things that I erased from my memories”); and (4) difficulties expressing or receiving gratitude (“I find it intimidating that someone can say “thanks” to me”).

### Intervention acceptability

Through Likert scale answers (1: not at all, 2: rather no, 3: rather yes, 4: absolutely), participants reported that the intervention was rather beneficial and had rather improved their quality of life, quality of relationship, meaning of life, psychological wellbeing, feeling of love or friendship, and the image they have of their relative (median: 3). They inferred that the intervention would be beneficial in the future (median: 3.5) and would absolutely recommend it to others (median: 4).

### Intervention effects

As shown in Table 3, we did not find any statistically significant change between T0 and T1 for patients. However, our quantitative results show a significant decrease in psychological distress for carers during the first phase of the study, in terms of depression (median = 3 at T0, 1.5 at T1, Z = -2.124, *p* = 0.034) and total score (median = 13 at T0, 7.5 at T1, Z = -2.045, *p* = 0.041), as shown in Table 4. We could not perform statistical analysis for carers in the second phase, as only 5 participated.

**Table 3:** Pre- and post-intervention quality of life, quality of relationship, psychological distress, and subjective burden for patients at phases 1 and 2

Patients	Median and interquartile range T0	Median and interquartile range T1	Statistical test—Z Wilcoxon	*P*-value
**Phase 1 (*n* = 11)**				
Quality of life (MQOL-R)				
Global (0–10)	7 (5–9)	7 (5–9)	-.427	.669
Physical (0–10)	5.7 (3.3–6.3)	5,7 (4–7)	-1.279	.201
Psychological (0–10)	6.5 (5.3–8.3)	5.3 (4.3–8.8)	-.714	.475
Existential (0–10)	7.5 (6.5–8.8)	7 (6.3–7.8)	-.666	.505
Relational (0–10)	9.7 (8.3–10)	8.7 (8.7–9.7)	-1.719	.086
Total score (0–10)	7.1 (6.2–8)	6.4 (5.8–7.2)	-.978	.328
Quality of relationship (PNRQ)				
Positive scale (0–48)	43 (39–47)	42.8 (38–47)	-.634	.526
Negative scale (0–48)	0 (0–1)	0 (0–2)	-.447	.655
Quality of relationship (CSI-4; 0–21)	21 (18–21)	20 (18–21)	-1.414	.157
Psychological distress (HADS)				
Depression (0–21)	7 (6–11)	6 (4–10)	-.655	.512
Anxiety (0–21)	6 (4–11)	7 (5–10)	-.104	.917
Total score (0–42)	14 (9–18)	15 (8–20)	-.535	.592
Subjective burden (SPBS; 0–50)	25 (21–32)	27 (24–32)	-1.179	.238
**Phase 2 (*n* = 12)**				
Grateful relationship (0–5)	5 (5–5)	5 (5–5)	.000	1.000
Satisfactory relationship (0–5)	5 (5–5)	5 (5–5)	-1.000	.317
Quality of life (0–10)	7 (4.3–9.5)	6 (4–10)	-.171	.865
Grateful feeling (1–5)	5 (4–5)	5 (4–5)	-.378	.705
Depression (0–10)	.5 (0–4.8)	0 (0–5)	-.816	.414
Anxiety (0–10)	3.5 (0–5.75)	1 (0–6)	-.271	.786
Subjective burden (0–10)	6 (0–8)	3 (0–5)	-.775	.438

**Table 4:** Pre- and post-intervention quality of life, quality of relationship, psychological distress, and subjective burden for carers at phase 1

**Carers (*n* = 11)**	**Median and interquartile range T0**	**Median and interquartile range T1**	**Statistical test—Z Wilcoxon**	***P*-value**
Quality of life (QOLLTI-F V2)				
Global (0–10)	6 (3–8)	6.5 (4.75–8.25)	-.954	.340
Environment (0–10)	9.5 (8–10)	8.3 (7–10)	-.422	.673
Patient condition (0–10)	2 (1–6)	4 (1.8–5.8)	-.719	.472
Carer’s own state (0–10)	6.2 (4.8–7.4)	7 (5.3–8.5)	-.816	.415
Carer’s outlook (0–10)	7.3 (6 -10)	7.5 (6.8–9.8)	-.341	.733
Quality of care (0–10)	9 (6.3–10)	8.3 (6.8–10)	-.170	.865
Relationships (0–10)	6.5 (4–8)	6.8 (5.5–9.3)	-.774	.439
Financial worries (0–10)	8 (2–10)	10 (1–10)	-.378	.705
Total score (0–10)	6.1 (5.9–7.2)	6.9 (6.1–8)	-1.172	.241
Quality of relationship (PNRQ)				
Positive scale (0–48)	38 (32–45)	39 (33–43)	-1.246	.213
Negative scale (0–48)	2 (1–9)	1.5 ( (0–9.2)	-.677	.498
Quality of relationship (CSI-4, 0–21)	15 (14–19)	15 (13.8–18)	-.853	.394
Psychological distress (BSI-18)				
Depression (0–24)	3 (2–7)	1.5 (0–4.5)	-2.124	.034
Anxiety (0–24)	6 (2–8)	2 (1–4.5)	-1.527	.127
Somatisation (0–12)	1 (1–4)	0.5 (0–1.8)	-1.292	.196
Panic (0–12)	2 (1–4)	1 (0–2)	-1.611	.107
Total score (0–72)	13 (7–20)	7.5 (1.8–12.3)	-2.045	.041
Subjective burden (BSFC-s; 0–30)	14 (8–18)	14 (6.8–16.3)	-1.177	.239

**Fig. 2:**
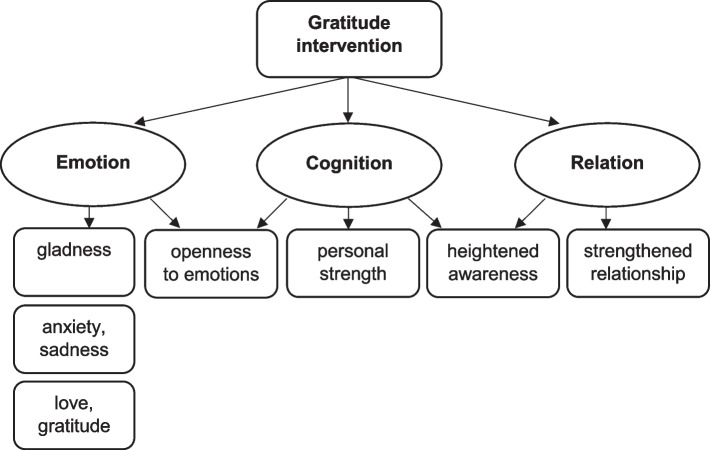
Thematic map of intervention effects

Thematic analysis of interviews indicates that overall, the intervention had: (1) multiple positive outcomes for 11/29 participants (5 patients, 6 carers), in the form of positive emotional and cognitive effects and, for five people, positive relational effects; (2) single positive outcomes for 14/29 participants (9 patients, 5 carers), in the form of either emotional or cognitive effects; (3) no outcome for two patients; and (4) negative outcomes for two patients, in the form of negative emotional effects. We identified seven main effects of the gratitude intervention, which are depicted in Fig. 2 and described below. As a methodological side note, we decided to include data on prevalence to our qualitative analysis in order to depict a precise picture of the intervention’s effects, provide rigorous information that could be relevant in terms of feasibility and acceptability of the intervention, and reinforce the mixed-methods character of our study. However, such results should not be used as a basis to draw inference about the prevalence of the effects depicted here beyond our study participants.

*Gladness.* When narrating their experience of the intervention, 21 interviewees spoke of the gladness – understood as “a positive emotional response to circumstances” [47] – they felt when remembering past experiences, writing or sharing letters. Thirteen people mentioned feeling “good”, “happy”, “glad” or “pleased” (“it did me good to tell him again […] that I’m really grateful”, partner-patient; “it was like when we receive a gift, it was nice, it was sweet”, daughter-carer). A patient described a physiological response to memories of the life he shared with his wife, as “a sensation of wellbeing that went down there on the belly.” Eight participants further highlighted that they felt “moved” or “touched” by the intervention, and five noted that expressing their gratitude in a letter made them “feel light” or “was liberating”.

*Anxiety, sadness.* The intervention triggered negative emotions in five participants, who felt anxious about writing (“I was afraid of staying in front of a blank page”, sister-carer) or sharing the letter (“to read it in front of him I was maybe scared to have all of a sudden floods of tears”, wife-carer). For most, anxiety soon receded (“it worried me before and after I was very happy”, mother-patient). However, two participants experienced particularly strong anxiety and sadness during the intervention (“I was in mourning, I felt sorrow, even about gratitude, because everything I wrote, it was in the past”, partner-patient). We offered professional support to participants who experienced such effects.

*Love and gratitude.* Taking part in the intervention led three participants to feel strong emotions of love and gratitude. A daughter-carer said that remembering her childhood with her mother “gives love into the heart”, whilst a husband-patient related: “I even shed some tears whist writing this gratitude letter and telling myself “[…] what a marvellous wife I have!” […] I was in a very very strong, very positive emotion of absolute gratitude and love”.

*Openness to emotions in everyday life.* Four people were inspired by the intervention to express or practice gratitude more broadly (“to have written this letter makes me want to be more into gratitude, in a general way”, healthcare professional). Others opened up to their emotions more generally, as a sister-carer explained: “it enables me to allow myself my emotions”.

*Personal strength.* A fourth effect relates to a perceived increase in personal strength or worth, which was mentioned by six participants. Some people remarked that the intervention gave them the strength “to keep going forward” (partner-carer) or helped them be “more serene towards some reactions” (partner-carer). Others felt more worthy of gratitude (“if I feel low, blue, […] I can tell myself “ah yes, my wife also thanked me for something”, husband-patient), or anticipated that the intervention might help them stay strong in the face of adversity (“by remembering the bond [with my mother], it can also help relationships afterwards, when everything goes wrong”, daughter-carer).

*Heightened awareness***.** Eight participants mentioned gaining greater awareness of the other’s support (“those were moments I lived that are memorable, hard, painful for me and writing them, I realised […] that I had a lot of help”, husband-patient), difficulties (“I thought about things I hadn’t thought of before […] in terms of what my husband is living through”, wife-carer), or gratitude (“since we did the letter, I realised that she was very grateful”, partner-relative). One patient further explained: “it made me understand a bit how I am on the inside […] in the illness and in pain”, which led him to “try to communicate differently when I feel I’m not well”.

*Strengthened relationship.* Five participants talked about the intervention as strengthening their relationship with their relative (“we were even better than before”, partner-carer; “we have this strength and we know it and we can lean on it and we know it’s shared”, husband-patient). For a carer, the intervention brought out a “much more altruistic, much stronger” dimension and “a truly strengthened bond” to his relationship with his partner.

## Discussion

### Intervention feasibility and acceptability

The aims of this study were to assess the feasibility and acceptability of a gratitude intervention for patients and carers and provide a preliminary assessment of its effects. Our results indicate good feasibility: eligibility rates were particularly encouraging; patient’s participation rate was comparable to other psychosocial and existential intervention studies [48–52] and improved in phase 2; carers’ participation rate was particularly high; and attrition rates were quite low for carers albeit high for patients, mainly because of progressing illness. Still, 82% of the patients who started the study completed it.

Some people were particularly interested in gratitude and regarded the intervention as a welcomed opportunity to express emotions they were already fully aware of. Others found it difficult to express themselves, struggled with physical difficulties (e.g. fatigue, limited hand mobility), or initially felt “unworthy” of gratitude. To overcome written expression and physical difficulties, gratitude interventions could rely on alternative forms of expression, such as recording a gratitude message, which enabled us to include people for whom writing represented a major obstacle.

Reasons for refusal to participate highlight a lack of interest in writing a gratitude letter in 35% of informed patients. Some found the research context “artificial” or not “spontaneous”, which seems to echo a general tendency to underestimate the potential positive impact of expressing one’s gratitude [53–55]. Moreover, the intervention completion rate of 7% of all assessed patients reflects broader difficulties in participant recruitment and retention in palliative care studies [56, 57], and raises questions about the representativeness of our participants, as further discussed below.

These findings, alongside notably heterogeneous modes of participation, expression, writing time, and transmission suggest that flexibility is key to ensure that the intervention is feasible and adapted to individual needs and preferences [58]. For instance, whilst we had initially planned to involve only dyads of patients and family carers, most patients chose to participate on their own when given the choice, perhaps to unburden their relatives, and three patients chose to perform the intervention with a healthcare professional in palliative care or oncology. Other key intervention features enhancing feasibility include its simplicity, low cost (as it does not require specific training), and flexibility (e.g. possibility to include both patients and carers, to write or audio-record a message).

### Intervention effects

Unlike other, larger scale gratitude intervention studies, we did not find quantitative correlations between our intervention and patients’ outcomes. We found a small but statistically significant post-intervention reduction in depression and psychological distress in carers, mirroring results from the wider literature on gratitude [59]. The general lack of statistically significant difference in participants before and after the intervention can be partly explained by high scores in most questionnaires at T0 (see Tables 3 and 4). This ceiling effect is rooted, in part, in a self-selection bias, as a majority of the individuals who agreed to participate were already “cultivating” or expressing gratitude, and described their relationship as good and open.

Still, in interviews, the narratives of most participants were studded with the difficulties that they faced in their everyday lives, which might be affecting their quality of life but were not captured by questionnaire results. This trend points to the limits of our quantitative assessment in capturing participants’ experiences, as noted by several people in interview (“your questionnaire had nothing to do with what I wrote”), suggesting that our choice of quantitative indicators was not optimal. Whilst our quantitative results are less encouraging than other gratitude intervention studies in oncology [15–17], the potential for comparison with this body of research remains limited insofar as our study and intervention designs differ from the latter (e.g. no control group, original gratitude intervention).

In this context, interviews were key to gain a better understanding of the experiences, perceptions, and relations of our participants. Qualitative analysis reveals that 25 out of 29 interviewees experienced positive emotional, cognitive and/or relational effects of the intervention. Qualitative data suggests that the intervention contributed – even if in a small or transient way – to emotional wellbeing, with a majority of people experiencing gladness at some point during the intervention, and to personal growth, as nearly half of our interviewees gained a sense of personal strength, gratitude, greater openness to their emotions, or a heightened awareness of the other’s experiences and support. This is aligned with results from studies in oncology, which found that gratitude interventions increased daily gratitude, daily self-esteem, and positive affects in participants [15, 16]. Such findings give credence to the broaden-and-build theory, which posits that experiencing positive emotions encourages people to broaden their horizons – or “thought-action repertoire” – through which they can build up new personal resources and experience more positive emotions, leading to a virtuous cycle of emotional wellbeing and resilience [60, 61].

Our qualitative results also suggest that the intervention improved the quality of the relationship of over a quarter of our interviewees, awaking feelings of love and gratitude or directly strengthening relationships. This echoes results from a study in which women with breast cancer who undertook gratitude journaling experienced a significant increase in perceived social support from their partners and others [15]. It also supports the suggestion that studies could consider relational elements alongside personal outcomes to more fully understand the effects of gratitude interventions [7].

Two patients noted overall detrimental effects of the intervention, in the form of anxiety and sadness. Both expressed distress thinking or speaking about their illness, suggesting that they faced difficulties living with severe illness. One person had to stop the interview after a few minutes because of the psychological distress she experienced when mentioning her illness. Indeed, this type of intervention can trigger difficulties and distress in participants, which are often not reported in the positive psychology literature but warrant particular attention. It is important for healthcare professionals wishing to suggest the intervention to their patients and / or their relatives to bear in mind that it can negatively affect participants, especially those for whom accepting life with severe illness is particularly challenging, and should not be proposed on a systematic basis.

In this light, a promising way to utilise the gratitude intervention would be to integrate it into individual therapy, an approach that has proven effective in improving the mental health of adults seeking psychotherapy [62]. Indeed, the quality of the therapeutic alliance between patients and their therapist has been associated with positive clinical outcomes [63, 64], and supported interventions were found to lead to greater effects than self-help ones [65]. Moreover, designing an intervention around the sole concept of gratitude is probably not optimal to reach people experiencing difficulties in their relationships, who might wish to express a mix of positive and negative emotions. Interventions that integrate both positive and negative emotions and experiences, which characterise the “second wave” of positive psychology, might be more effective in reaching and supporting people with personal and relational difficulties [66, 67].

### Limitations and perspectives

This study has several limitations. First of all, as this is a pilot and feasibility study characterised by low statistical power and without a control group, it is not possible to interpret our results in terms of intervention effectiveness or to transpose them to different contexts. It would be particularly interesting for future studies to consider gratitude interventions in different contexts worldwide to explore their relevance and potential effects across a variety of socio-cultural settings – as Tan and colleagues have done in Indonesia for instance [17]. Secondly, the above-discussed self-selection bias was exacerbated by our study’s eligibility criteria, which led to the exclusion of unstable and socially isolated patients, and of those with cognitive or psychiatric disorders and severe communication issues. As such, our patient-participants were not representative of the general palliative patient population, which begs the question of how to maximise inclusivity in palliative care psychosocial studies without compromising data quality. Thirdly, we did not consider the medium to long term effects of the gratitude intervention in participants, which future research could explore. Indeed, research suggests that introspective letter writing processes combined with the social and behavioural aspects of sharing one’s thoughts and feelings might be key to delivering lasting positive psychological effects [68, 69]. Fourthly, since most participants both wrote and received a gratitude letter, we could not investigate the specific effects of different activities (i.e. writing, sharing, receiving the letter). Finally, this article does not present an analysis of the letters’ content, which will be the subject of a future article.

## Conclusions

This study has shown that our gratitude intervention, based on writing and sharing a gratitude letter, is feasible and acceptable. Qualitative analysis has highlighted beneficial effects on the majority of participants in terms of emotions, cognition and, for some, relationships. Larger scale deployment and evaluation of the gratitude intervention, including a control group, is warranted in order to have a more reliable evaluation of its effectiveness in the palliative care setting. To do so, quantitative indicators could be adapted to capture the effects identified through qualitative analysis. A simple quantitative design in the form of a short questionnaire would also ensure minimum burden on participants, which might help improve retention rates. Furthermore, identifying and including patients and carers experiencing relational or emotional distress might help to better understand the potential impact of the intervention and improve participants’ representativeness. Finally, the intervention should adopt flexible modalities (as in phase 2 of this study), whereby people could chose to participate on their own or with a person of their choice, and to write, dictate, or record their message.

Based on our data, we believe that the integration of a gratitude-based approach into individual therapy may offer the greatest potential for clinical applicability and usefulness. We will explore this hypothesis in a future study.

## Supplementary Information

**Additional file 1.** Instructions for gratitude intervention.

**Additional file 2.** Semi-structured interview guide.

## Data Availability

The datasets analysed during the current study are available from the corresponding author on reasonable request.

## Abbreviations

BSCF-s: Burden Scale for Family Caregivers—short version
BSI-18: Brief symptom inventory-18
CSI-4: Couple Satisfaction Index,
GAC: Gratitude Adjective Checklist
HADS: Hospital Anxiety and Depression Scale
IPOS: Integrated Palliative Care Outcome Scale
MQoL-r: McGill Quality of Life Questionnaire – Revised
PN-RQ: Positive–Negative Relationship Quality scale
QOLLTI-F V2: Quality of Life in Life-Threatening Illness—Family Carer Version 2
SPBS: Self-perceived Burden Scale
