# Supplementary material for: Giving and receiving thanks: a mixed methods pilot study of a gratitude intervention for palliative patients and their carers
Source: BMC Palliat Care. 2023 Apr 26;22:52. doi: 10.1186/s12904-023-01172-x (PMC10134658; doi:10.1186/s12904-023-01172-x)
Supplement: Supplementary file 1 — Additional file 1. Instructions for gratitude intervention. [file 12904_2023_1172_MOESM1_ESM.docx]

**Additional file 1** Instructions for gratitude intervention

Includes instructions for study participants on writing their gratitude letter.

Most people appreciate being thanked for a job well done or for a favour done for a friend, and most of us remember having thanked someone at some point. However, our “thanks” is sometimes so spontaneous and automatic that it becomes almost meaningless. In this exercise, you will have the opportunity to express your gratitude in a more thoughtful way.

First, take a moment to think back about the past few years and remember when _[your relative]_ did something for you, for which you feel extremely grateful. Think about the impact that he/she had on your life.

Now, take a moment to write a letter to this person.

Next, and only if you wish to do so, you will have the possibility to read her/him the letter, to ask her/him to read it in front of you, or to let her/him read it alone.

Use the instructions below to help you complete this task:

1. Use whichever letter format you like, but remember to write as if you were directly talking to the person you feel grateful towards.

2. Don’t worry about spelling and grammar.

3. Describe specifically why you are grateful towards this person and how her/his behaviour has impacted your life.

4. Describe what you are doing now and how you remember her/his efforts.

5. The letter should be approximatively one page long.

***Remember****: Even though, as part of the study, you will show or give this letter to someone, the letter you are writing is a private document in which you can freely express your gratitude. Everything that you write will remain strictly confidential, unless you give your permission to a researcher to retain a copy of your letter for the purpose of this study. In this case, it will be identified by a number, not by your name.*
