# Supplementary material for: Giving and receiving thanks: a mixed methods pilot study of a gratitude intervention for palliative patients and their carers
Source: BMC Palliat Care. 2023 Apr 26;22:52. doi: 10.1186/s12904-023-01172-x (PMC10134658; doi:10.1186/s12904-023-01172-x)
Supplement: Supplementary file 2 — Additional file 2. Semi-structured interview guide. [file 12904_2023_1172_MOESM2_ESM.docx]

**Additional file 2** Semi-structured interview guide

Includes questions used in semi-structured interviews with study participants

| Theme | Objective | Main question |
| --- | --- | --- |
| **Personal representations and experiences of gratitude** | Understanding the personal definition and meaning of gratitude | **What does the term 'gratitude' mean to you?** |
|  |  | To investigate |
|  |  | What are your personal experiences with gratitude?  Can you give me some examples?  Do you usually express gratitude?  If so, how do you usually express your gratitude?  What do you generally feel gratitude towards (sources of gratitude)? |
| **Understanding the intervention** | Understanding what participants take away from this intervention | Main question |
|  |  | *We will now look back at what you have experienced during this intervention...*  **What did you remember particularly vividly from the exercise?** |
|  |  | To investigate |
|  |  | What do you remember from this exercise / What did you understand from this exercise / What was the focus of this exercise for you?  What did it mean to you in terms of the role you played (e.g. writing/transmitting vs. receiving)? |
| **Feelings/experiences about the intervention** | Understanding how participants experienced the different "parts" of the intervention (writing, transmission, reception) | Main question |
|  |  | **Can you tell me how it all happened?** |
|  |  | To investigate |
|  |  | WRITING  *Can you tell me what it was like to write the letter...*  1. How did you feel about the prospect of writing the letter (before writing)?  2. How did you feel when you wrote it?  3. How did you feel after writing it?  TRANSMISSION  *Can you tell me how you experienced transmitting the letter?*  1. How did you choose to send the letter?  2. 2. Why?  3. How did you feel before transmitting it?  4. What about when you transmitted it?  5. How did you feel after transmitting it?  RECEPTION  *Can you tell me what it was like for you to receive the letter...*  How did you feel at that time?  How did you feel after receiving it? |
| **Barriers and facilitators** | Understanding what was easy and what was more complicated in undertaking the intervention | Main question |
|  |  | **What was difficult for you, in this exercise?**  **Conversely, what did you find easy?** |
|  |  | To investigate |
|  |  | For writing - For transmission - For reception  What helped you?  What obstacles did you encounter? |
| **Impact(s)** | Assessing the adequacy between the changes reported by participants and quantitative outcomes (quality of the relationship, quality of life, psychological distress, subjective burden) | Main question |
|  |  | **We did this study to find out if this intervention (the letter exercise) has an impact for people in the same situation as you. What are your thoughts on this?** |
|  |  | To investigate |
|  |  | How has this exercise changed you/what impact or influence did it have for you?   - on which areas of your life? - at the individual level ? and at the relationship level ?   Do you find that the themes in the questionnaires (quality of life, quality of relationship, subjective burden, anxiety/depression) echoed / were consistent with the changes you may have experienced? |
